# Exploring the Combined Effect of Bm86 and Subolesin Polypeptide Vaccines in Cattle Naturally Infested with *Rhipicephalus microplus*

**DOI:** 10.3390/vetsci13030301

**Published:** 2026-03-22

**Authors:** Nancy Mendoza-Martínez, Miguel Ángel Alonso-Díaz, Jose Octavio Merino-Charrez, Rodolfo Lagunes-Quintanilla

**Affiliations:** 1 Facultad de Medicina Veterinaria y Zootecnia, Universidad Nacional Autónoma de México, Avenida Universidad 3000, Ciudad de México 04510, Mexico; 2 Centro de Enseñanza, Investigación y Extensión en Ganadería Tropical, Facultad de Medicina Veterinaria y Zootecnia, Universidad Nacional Autónoma de México, Km. 5.5 Carretera Federal Tlapacoyan-Martínez de La Torre, Martínez de La Torre 93600, Mexico; 3 Facultad de Medicina Veterinaria y Zootecnia, Universidad Autónoma de Tamaulipas, Km. 5, Carretera Victoria Mante, Ciudad Victoria 87000, Mexico; 4 Centro Nacional de Investigación Disciplinaria en Salud Animal e Inocuidad-INIFAP, Carretera Federal Cuernavaca-Cuautla 8534, Col. Progreso, Jiutepec 62550, Mexico

**Keywords:** *Rhipicephalus microplus*, pBm86, pSubolesin, cocktail vaccine, cattle

## Abstract

*Rhipicephalus microplus* infestations are a major concern for cattle production in neotropical regions. Anti-tick vaccines represent a sustainable alternative to chemical acaricides; however, improving vaccine efficacy remains challenging. This study evaluated the protective efficacy of polypeptide-based formulations of pBm86 and pSubolesin administered as co-immunization or as a cocktail in naturally infested cattle. Twelve calves were assigned to three groups: G1: 100 μg pBm86 + 50 μg pSubolesin co-immunized; G2: same formulation in cocktail; and G3: control. The cocktail significantly reduced adult female tick numbers, oviposition, and larvae hatching (*p* < 0.05), achieving a vaccine efficacy of 75%. In contrast, co-immunization achieved 50% efficacy. Vaccinated cattle developed significantly increased antigen-specific IgG levels against Subolesin than Bm86. IgG antibodies recognized native tick proteins by Western blot, and their efficacy was confirmed in vitro using blood-feeding assays. These results indicate that the cocktail formulation showed improved efficacy compared with co-immunization in this study, possibly by affecting complementary biological processes in ticks. Finally, this study supports the development of multi-antigen, polypeptide-based vaccines as a promising and sustainable approach for the control of *R. microplus* under natural field conditions.

## Simple Summary

Cattle tick (*Rhipicephalus microplus*) is a major problem for cattle production in tropical and subtropical regions, where control often relies on chemical treatments that can be costly and may lose effectiveness over time due to the development of resistant tick populations. Vaccination is considered a sustainable alternative to chemical acaricides; however, improving vaccine efficacy remains a challenge. In this study, we evaluated a vaccine formulation combining two tick antigens, pBm86 and pSubolesin, in naturally infested cattle. The combined formulation reduced tick numbers and negatively affected tick reproduction, showing improved performance compared with the co-immunization group within this study. Vaccinated cattle developed significantly increased antigen-specific IgG levels, particularly against Subolesin. These results advance the development of cocktail vaccines to control *R. microplus* ticks and support further evaluation of multi-antigen vaccines as part of integrated tick management strategies to improve tick control and promote sustainable cattle production.

## 1. Introduction

*Rhipicephalus microplus* is the most significant ectoparasite affecting livestock in tropical and subtropical regions. It has direct negative effects on animal health, including decreased weight gain, reduced milk production, and hide damage, as well as indirect effects through the transmission of babesiosis and anaplasmosis [1]. The infestation of *R. microplus* has a substantial impact on cattle production across the Americas, Africa, and Southeast Asia [2], resulting in economic losses estimated at approximately US$ 22–30 billion annually [3]. Furthermore, recent studies suggest that climate change has contributed to the expansion of tick geographic ranges, thereby increasing the risks to animal health and productivity and posing a growing threat to the global cattle industry [2].

The main strategy for controlling *R. microplus* has relied on the intensive, continuous use of chemical acaricides for decades [1,4]. However, this approach has led to several serious drawbacks, including the emergence of multi-resistant tick populations, environmental contamination, and concerns related to food safety and security [4].

These issues underscore the urgent need for alternative, sustainable strategies to control tick infestations, such as anti-tick vaccines that can be incorporated into integrated tick management (ITM) programs [1]. Currently, the only commercially available anti-tick vaccine is based on the Bm86 antigen, which has shown variable efficacy against tick infestations worldwide due to genetic polymorphisms in the target protein across different geographic regions and tick populations [5,6]. Consequently, several research groups have focused on identifying and evaluating novel tick antigens and multi-antigen vaccine formulations to improve protective efficacy and expand the applicability of vaccines beyond single-antigen approaches [7].

A particularly promising tick antigen is Subolesin, a highly conserved protective antigen across multiple tick species and other arthropods that regulates gene expression associated with tick development, reproduction, and the immune response [8,9]. Immunization trials in cattle with this antigen have reported an efficacy of 60% or higher against *R. microplus* [10,11] and, more recently, over 90% in Uganda using a region-specific vaccine approach targeting *R. appendiculatus* and *R. decoloratus* [12]. Nevertheless, evaluating novel strategies to enhance the efficacy of different antigenic targets in cattle trials remains imperative.

One approach to improving anti-tick vaccine performance was proposed by Willadsen [13], who demonstrated that combining two or more antigens can increase vaccine efficacy compared to immunization with a single antigen. Accordingly, several multi-antigen vaccination strategies, including antigen cocktails, co-immunization, and chimeric antigens, have been explored in anti-tick vaccine research. These approaches have been tested in different combinations, resulting in synergistic or antagonistic effects [11,14,15]. This variability highlights the complexity of selecting appropriate antigen combinations for effective multi-antigen vaccines, as antigenic competition is a significant challenge [16]. Furthermore, the limited number of studies conducted under field conditions further complicates the translation of these strategies into practical applications.

The combination of Bm86 and Subolesin has been previously evaluated in cattle trials. An immunization study conducted under controlled conditions, using a vaccine cocktail containing Bm86 (50 μg) and Subolesin (50 μg) per dose, administered in a three-dose regimen, reported an overall efficacy of 23% against *R. microplus* [unpublished data]. Another study reported 97% efficacy in reducing the number of *R. microplus* ticks after administering 100 μg of each antigen in a three-dose regimen under controlled conditions [17]. However, these results have not been consistently replicated using this antigen combination. Moreover, several immunization trials have demonstrated antigenic competition when these antigens are combined [11,18]. Recently, our research group designed and characterized polypeptides derived from Bm86 and Subolesin as alternative vaccine candidates [19,20]. This strategy aims to replace the use of full-length proteins, which often present limitations related to the molecular size, structural complexity, potential cross-reactivity, or immunogenicity [7]. In this way, important questions remain about the optimal formulation, delivery strategy, and antigen combination required to enhance vaccine efficacy while minimizing antigenic competition. Therefore, this study aims to evaluate the efficacy of some formulations containing Subolesin and Bm86 polypeptides, administered either as co-immunization or as a cocktail, on the reproductive performance of *R. microplus* in naturally infested cattle.

## 2. Materials and Methods

### 2.1. Study Location and Animals

The study was carried out at the Center for Teaching, Research, and Extension in Tropical Livestock (CEIEGT) of the Faculty of Veterinary Medicine and Zootechnics of the National Autonomous University of Mexico (FMVZ-UNAM), located in Martínez de la Torre, Veracruz, Mexico (20°1′ N, 97°6′ W; 151 masl). The climate of this location is classified as humid tropical, featuring three distinct seasons: a rainy season from June to September, a winter season from October to January, and a dry season from February to May. The average annual temperature is 23.4 °C, with a mean relative humidity of 85% and an annual rainfall of 1743 mm [21]. The experiment was conducted for 24 weeks, from May to October 2023. Twelve female calves F1 (Holstein × Zebu) (13 ± 0.5 months; 184 ± 18 kg), with prior exposure to *R. microplus,* were used. The animals were clinically healthy and managed under a rotational grazing system on *Urochloa brizantha* and native grasses, supplemented with commercial concentrates, and had *ad libitum* access to water. Before and throughout the immunization trial, the pastures naturally harbored *R. microplus* infestations, ensuring that observed infestations reflected the natural field conditions. Over the past three months, the animals were not treated with acaricides and were closely monitored by a veterinarian for babesiosis and anaplasmosis during the experiment. Cattle were maintained and handled following the guidelines and protocols approved by the Internal Sub-Committee for Care and Use of Experimental Animals from FMVZ-UNAM.

### 2.2. Antigen Production and Vaccine Formulation

The recombinant polypeptide Bm86 (pBm86) and the polypeptide Subolesin (pSubolesin) were produced in *Escherichia coli* BL21 Star (DE3), purified to >90% for vaccine formulation, and adjuvanted with Montanide ISA 50 V2 (Seppic, Puteaux, France), as previously described [19,22]. Vaccine formulations, including co-immunization and cocktail preparations, were prepared at a final volume of 2 mL/dose.

### 2.3. Immunization Trial

**Figure 1:**
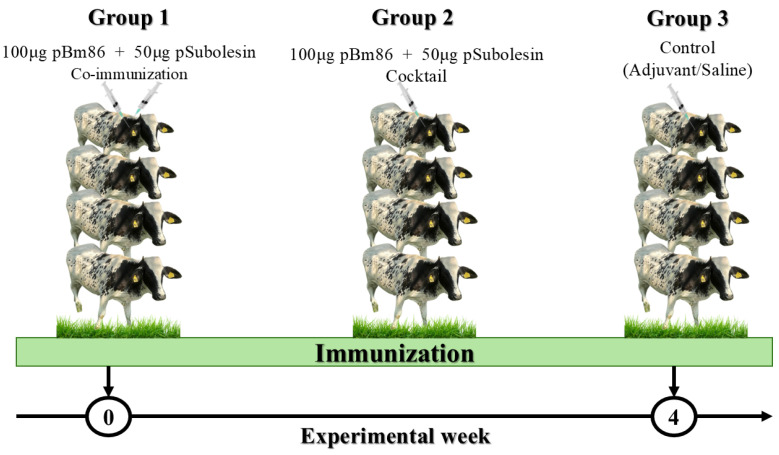
Immunization scheme in cattle naturally infested with *Rhipicephalus microplus*.

Cattle were randomly assigned to three experimental groups, each comprising four animals. Group 1 was immunized with 100 μg pBm86 and 50 μg pSubolesin administered as co-immunization, indicating that each recombinant antigen was injected separately at different anatomical sites on the right and left sides of the neck. Group 2 was immunized with 100 μg pBm86 and 50 μg pSubolesin, combined and administered as a cocktail, injected in a single shot on the neck. Group 3 received phosphate-buffered saline (PBS) formulated with Montanide ISA 50 V2 as a control. All animals were subcutaneously immunized using 5 mL syringes with an 18 G needle in experimental weeks 0 and 4. Animals were routinely monitored by a veterinarian for local injection site reactions and for any clinical abnormalities after immunization (Figure 1).

### 2.4. Tick Data Collection and Evaluation

Cattle were kept in the same tick-infested paddocks, naturally infested with *R. microplus*, for three months before the study and throughout the experimental trial. To evaluate the effect of vaccination on tick biology, adult female ticks (4.5–8 mm) were collected weekly from one side of the animal and multiplied by two to estimate the total number of adult ticks per animal [23]. All collected ticks were counted, individually weighted, and incubated in Petri dishes at 27 °C and 80% relative humidity to allow oviposition and larvae hatching. Vaccine efficacy was measured by the reduction in adult female ticks, tick weight, oviposition, and larvae hatching. The percentage reduction was calculated with respect to the control group using the standardized formula [24].

### 2.5. ELISA Antibody Serology

Before each immunization and every 15 days for 24 weeks, blood samples were collected via the caudal vein into sterile tubes without anticoagulant and maintained at 4 °C until arrival at the laboratory. The serum was then separated after centrifugation and stored at −20 °C until further analysis by indirect ELISA. Purified pBm86 and pSubolesin antigens (0.1 μg/well) were used to coat ELISA plates overnight at 4 °C. Subsequently, the wells were washed three times using PBS + 0.05% Tween 20 (PBS-Tw), blocked with PBS-Tw + 5% skim milk, and incubated for 1 h at room temperature. Following three additional washes with PBS-Tw, the plates were incubated with sera samples diluted to 1:100 in PBS-Tw for 1 h at room temperature, followed by a 1:2000 dilution of conjugated anti-bovine IgG-AP (Sigma-Aldrich, St. Louis, MO, USA). The color reaction was developed with p-nitrophenyl phosphate (pNPP) (Sigma-Aldrich, St. Louis, MO, USA), and the optical density (OD) was read at 405 nm. The co-immunization and cocktail vaccine results were evaluated independently for each antigen. Antibody levels between the immunized groups and the control group were compared by the Kruskal–Wallis test (*p* < 0.05).

### 2.6. Detection of Native Protein in R. microplus Tissues by Western Blot

Five engorged *R. microplus* female ticks were collected and washed with PBS. The dorsal cuticle was dissected with a scalpel, and the salivary glands, guts, and ovaries were carefully separated and removed using fine forceps. These tissues were washed twice in PBS and stored at −70 °C until further use. The extracts were prepared following the method described by Popara et al. [25]. Protein concentrations were determined using the Bradford assay with bovine serum albumin as the reference standard. Subsequently, protein extracts were electrophoresed using 15% sodium dodecyl sulfate–polyacrylamide gel electrophoresis (SDS-PAGE) gels and transferred onto polyvinylidene difluoride (PVDF) membranes. The PVDF sheets were blocked in PBS-Tw + 5% skimmed milk and incubated for 1 h at 4 °C with gentle shaking. The membranes were washed three times in PBS-Tw, cut into strips (~0.4 mm), and incubated for 2 h at 4 °C with serum diluted to 1:300 in PBS-Tw from a representative animal from each group collected a week after the second immunization. Then, the strips were washed as previously described and incubated with an anti-bovine IgG-AP conjugate (Sigma-Aldrich, St. Louis, MO, USA) diluted to 1:2000 in PBS-Tw for 2 h at room temperature. Finally, the positive signal was revealed using a BCIP/NBT alkaline phosphatase substrate (Millipore, Bedford, MA, USA).

### 2.7. Tick Capillary Feeding

The capillary feeding assay was conducted to assess the functional impact of vaccine-induced IgG antibodies on tick weight and oviposition under controlled in vitro conditions. A six-month-old bovine *Bos taurus* with no prior history of tick infestations and no acaricide treatments was artificially infested with *R. microplus* “Media Joya” tick strain larvae (belonging to the CENID-SAI, INIFAP, Morelos, Mexico). Subsequently, partially engorged *R. microplus* ticks were recovered manually 20–21 days post-infestation. Then, 25 mL of blood was obtained from a bovine with no history of immunization into sterile tubes containing 3.2% sodium citrate anticoagulant. IgGs were purified using the Montage Antibody purification kit and PROSEP-A spin columns (Millipore, Bedford, MA, USA) according to the manufacturer’s recommendations and mixed with sodium citrate-treated bovine blood at a concentration of 1 mg/mL of preimmune or antigen-specific purified IgGs from one representative calf of each group. Afterward, the ticks were cleaned, weighed, and fixed on glass plates (150 mm × 110 mm) using double-sided adhesive tape (3 M, St. Paul, MN, USA). Females were discarded if they had damage in the hypostome or if their weight did not lie between 25 and 60 mg. Ticks were divided into three experimental groups, each consisting of 12 individuals, and fed for 48 h. Microhematocrit capillary tubes (75 mm × 1.0 mm, ∅1.5 mm) were filled with the blood meal prepared as described above and placed over the ticks’ mouthparts. Tubes were replaced every 2–3 h, as described by Antunes et al. [26]. After feeding, ticks were detached from the double-sided tape and weighed again to determine the amount of blood ingested. Female ticks were placed in Petri dishes and incubated at 27 °C and 80% relative humidity for oviposition [Supplementary Figure S1]. Student’s *t*-test for unequal variance compared tick weight increases during feeding and oviposition (*p* < 0.05).

### 2.8. Environmental Data Collection

Eleven days before and during the experimental trial, the environmental temperature (C°) and relative humidity (%) were recorded daily. The data were obtained through The Weather Channel© mobile app version 14.27. The average monthly data for the two environmental parameters were calculated. Total rainfall was obtained through the National Weather Service database [27].

### 2.9. Statistical Analysis

Data were statistically analyzed using StatGraphics software, version 19.1.3. The Kruskal–Wallis test was used to compare the results of tick number, tick weight, oviposition, and larvae hatching between the immunized and control groups. The results are presented as average ± standard deviation (SD), and differences were considered statistically significant when *p* < 0.05. A correlation analysis was conducted to compare the number of ticks collected after feeding with the antibody level measured after the second immunization through the end of the experiment, using Spearman’s test (*p* < 0.05). Additionally, monthly rainfall records and monthly averages of environmental temperature and relative humidity were correlated with tick infestations using Pearson’s test (*p* < 0.05).

## 3. Results

### 3.1. Effect of Immunization on Biological Parameters of R. microplus Ticks

The biological parameters of engorged female *R. microplus* ticks collected from naturally infested cattle are shown in Table 1. The immunization effect in Group 1 was significant for the larvae hatching parameter compared with the controls (*p* < 0.05). Group 2 showed significant differences in adult female tick numbers, oviposition and larvae hatching parameters compared with the control group (*p* < 0.05). Based on effects on tick number and reproductive parameters, the calculated overall vaccine efficacies were 50% and 75% for Groups 1 and 2, respectively.

### 3.2. Immune Response

**Figure 2:**
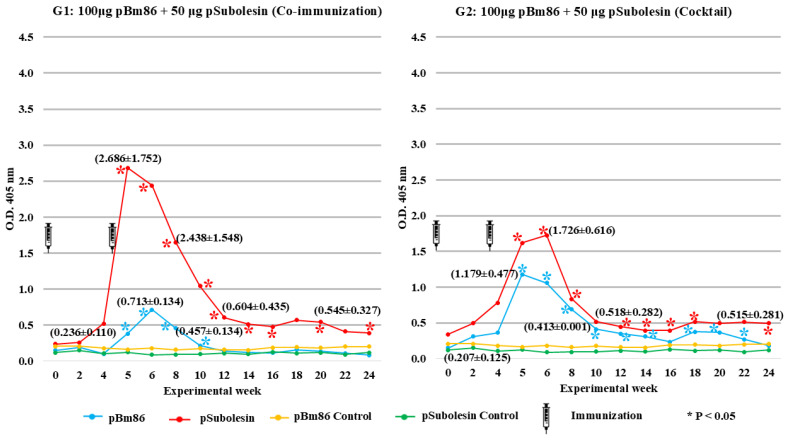
Indirect ELISA determined bovine serum antibody levels in immunized and adjuvant/saline control groups for cattle. Antibody levels in immunized cattle were expressed as the OD_405nm_ value for the serum dilution (1:100). Average ± SD values are shown in parentheses, as well as average comparison between immunized and control cattle using a Kruskal–Wallis test (* *p* < 0.05; N = 4). Black syringes indicate immunogen application.

The humoral immune response induced in cattle is shown in Figure 2. IgG levels increased in the immunized groups after the second immunization compared with the control group (*p* < 0.05). Both experimental groups developed higher antibody levels against pSubolesin than against pBm86. The IgG anti-pSubolesin antibodies reached maximum values of 2.7 OD and 1.7 OD units in Groups 1 and 2, respectively, while the highest values for pBm86 antibodies were 0.7 OD and 1.1 OD in Groups 1 and 2, respectively. Although antibody levels decreased for both antigens from week 8 to the end of the experiment, IgG levels against pSubolesin and pBm86 showed significant differences in both experimental groups compared with the control group (*p* < 0.05).

### 3.3. Correlation Between IgG Antibody Levels and Tick Infestation

**Figure 3:**
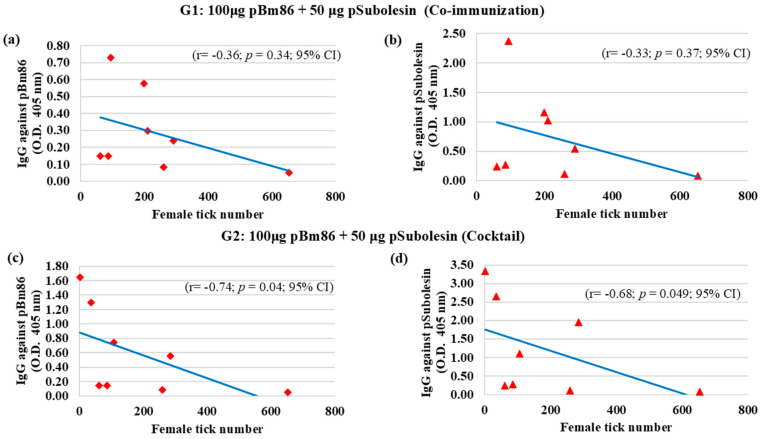
Spearman’s Rho correlation analyses (*p* < 0.05) were conducted to correlate the anti-pBm86 and anti-pSubolesin IgG levels in immunized cattle with the adult female tick number collected during the experimental trial in individual cattle (N = 8). (**a**) Group 1: anti-pBm86 IgG; (**b**) Group 1: anti-pSubolesin IgG; (**c**) Group 2: anti-pBm86 IgG; (**d**) Group 2: anti-pSubolesin IgG. The linear correlation coefficient (r), *p*-value, and confidence interval (CI) are shown.

The level of IgG antibodies after the second immunization (week 5) until the end of the experiment (week 24) was correlated with the number of ticks collected after feeding. In Group 1, no significant correlation was observed between IgG antibody levels induced by pBm86 (r = −0.36; *p* = 0.34, CI 95%) or pSubolesin (r = −0.33; *p* = 0.37, CI 95%) and tick infestation levels (Figure 3a,b). In contrast, Group 2 showed a negative correlation between IgG antibody levels and tick infestation, with r = −0.74 (*p* = 0.04; CI 95%) for anti-pBm86 and r = −0.68 (*p* = 0.049; CI 95%) for anti-pSubolesin (Figure 3c,d). These results suggest that higher IgG antibody levels in immunized cattle were associated with a greater reduction in the number of ticks collected.

### 3.4. Western Blot Analysis

**Figure 4:**
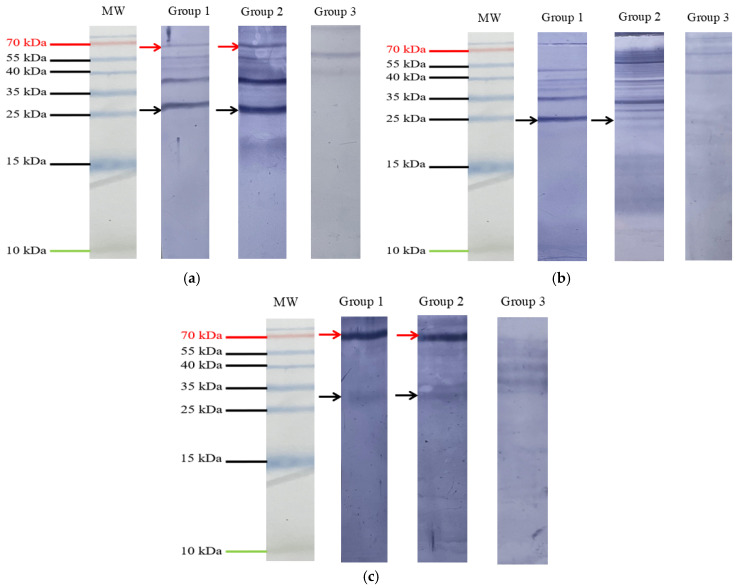
Western blot analysis of tick tissues. Ten micrograms of (**a**) ovary, (**b**) salivary gland, and (**c**) gut tissue sections were loaded onto SDS-PAGE gels and transferred to PVDF membranes. Sera from one representative calf per group: Group 1 (co-immunized), Group 2 (cocktail), and Group 3 (control) were used. Hybridization signals were developed with an anti-bovine IgG-AP conjugate. Native Bm86 proteins are indicated with red arrows, and native Subolesin proteins are indicated with black arrows. MW, molecular weight.

Western blot analysis showed that sera collected a week after the second immunization from immunized cattle recognized the native Bm86 and Subolesin proteins compared to the controls (Figure 4). The anti-pSubolesin IgG antibodies from a representative calf recognized the native protein derived from *R. microplus* female ovaries and salivary glands in immunized groups (Figure 4a,b). Additionally, the native Bm86 was strongly detected in gut extracts from both immunized groups (Figure 4c) and in ovarian tissues from Group 2 (Figure 4a). In contrast, Group 1 showed low detection of the native Bm86 in ovarian tissues (Figure 4a) and low immunoreactivity to the native Subolesin in the gut extracts (Figure 4c). No immunoreactive bands were detected when membranes were incubated with sera from the control group. Bands at ~25 and ~72 kDa were consistent with Subolesin and Bm86, respectively. Additional bands of varying molecular weights were also detected in tick tissues. These bands are likely associated with antibodies produced before the immunization trial due to prior tick exposure and may recognize other tick proteins.

### 3.5. Effect of Anti-pBm86 and Anti-pSubolesin Antibodies on Tick Weight and Oviposition In Vitro

**Figure 5:**
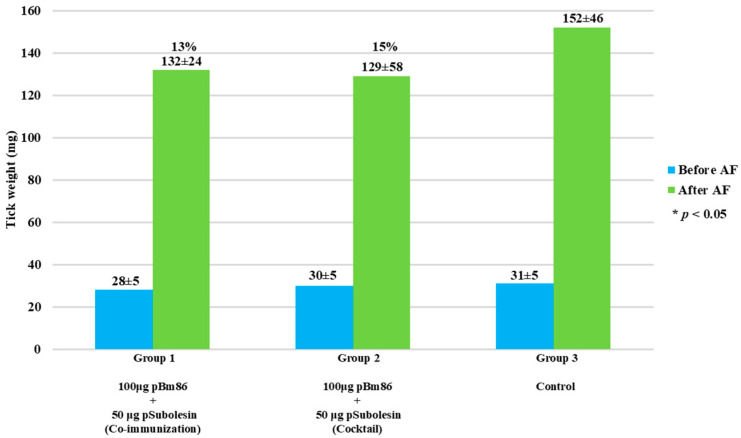
Effect of antibodies on tick weight. Ticks (N = 12) were weighed before and after capillary feeding. The percent reduction was calculated with respect to the control group and compared using Student’s *t*-test (* *p* < 0.05). In parentheses, the average ± SD values are shown. AF: artificial feeding.

**Figure 6:**
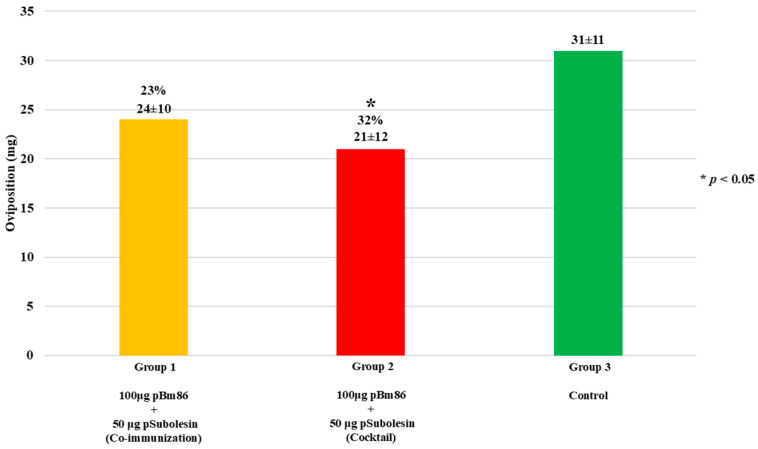
Effect of antibodies on tick oviposition. Ticks (N = 12) were incubated for oviposition after capillary feeding, and the percent reduction in egg production was calculated with respect to the control group and compared using Student’s *t*-test (* *p* < 0.05). In parentheses, the average ± SD values are shown.

Capillary feeding was performed to evaluate the effect of antibodies against ticks fed on blood supplemented with anti-Bm86 IgG and anti-pSubolesin on tick weight and oviposition in vitro. After 48 h of feeding, there was no statistical difference in tick weight compared to control ticks, with weight reductions of 13% and 15% for Groups 1 and 2, respectively. Nevertheless, a consistent trend was observed, as ticks from the control group showed the highest weight gain during the feeding period (Figure 5). In contrast, a significant reduction in oviposition was observed in ticks fed with sera from Group 2, showing a reduction of 32% in egg production compared with the control group (*p* < 0.05) (Figure 6). It is noteworthy that several ticks failed to lay eggs in both immunized groups.

### 3.6. Environmental Factors and Correlation with Tick Infestation

Monthly averages of environmental parameters, including temperature, relative humidity, and total rainfall (mm), are presented in Supplementary Table S1. Monthly environmental temperature ranged from 27.3 °C to 29.5 °C, and relative humidity ranged from 70.6% to 78.9%. Monthly total rainfall ranged from 23.9 mm to 533.2 mm. From June to August, a progressive increase in the number of adult engorged female *R. microplus* ticks was observed, reaching a peak in September (Supplementary Figure S2a). However, the analysis showed no significant correlation between tick infestation and environmental variables, including temperature (R^2^ = −0.12; *p* = 0.82, 95% CI), RH (R^2^ = −0.46; *p* = 0.36, 95% CI), or rainfall (R^2^ = −0.08; *p* = 0.88, 95% CI), throughout the experimental trial (Supplementary Figure S2b–d).

## 4. Discussion

Tick vaccines based on the Bm86 and Subolesin antigens have been studied as a proposed cocktail approach against *R. microplus*. However, the results differed from those expected, possibly due to synergistic or antagonistic interactions between the antigens when combined [11,17,18]. The tests reported herein were designed to standardize doses capable of eliciting an effective immune response through two strategies (co-immunization and cocktail) to determine which strategy reduces *R. microplus* tick populations in cattle. The concentrations of the pBm86 and pSubolesin antigens used in this study were selected based on previous field studies [11,22]. Here, a formulation consisting of 100 μg pBm86 + 50 μg pSubolesin administered as a cocktail resulted in enhanced control of *R. microplus* in naturally infested cattle. Our results indicate a significant reduction in the number of female ticks (59%), oviposition (20%), and larvae hatching (25%) compared with the control group (*p* < 0.05), resulting in an overall efficacy of 75%. The immunized cattle developed a significant antigen-specific humoral immune response. Following the second immunization, high anti-pBm86 IgG and anti-pSubolesin levels were observed and remained significantly higher than in the control group throughout the experiment (*p* < 0.05). Furthermore, a significant negative correlation was observed between IgG antibody levels against both antigens and tick infestation levels in this group, indicating that higher antibody levels were associated with reduced tick infestation, as previously reported [6,9,10,11,19,22]. These findings are higher than those reported in other studies using the Bm86 and Subolesin antigens, either as a single antigen or in a cocktail [6,9,10,17,18,19,23,24], suggesting that IgG antibodies generated in cattle reduce reproductive parameters in *R. microplus* ticks.

A greater protective effect was observed with the cocktail formulation at the evaluated concentration compared with previously reported single-antigen approaches using pBm86 or pSubolesin under comparable field conditions, which showed efficacies of 58% and 67%, respectively [11,22]. However, cross-study comparisons should be interpreted cautiously due to potential differences in environmental conditions, host factors, and experimental design. This observation is similar to prior research identifying immunoprotective effects against *Rhipicephalus* spp. in in vitro trials. These studies demonstrated that silencing the expression of Subolesin and Rs86 (the Bm86 homolog in *R. sanguineus*) using iRNA reduced tick attachment, feeding, and oviposition [28]. The experiments reported herein on capillary feeding suggest that antibodies generated against pBm86 and pSubolesin reveal distinct mechanisms in tick biology and may enhance efficacy when administered in a cocktail. Although no statistically significant reduction in tick weight was observed, a notable reduction of 32% in egg production was documented. These results are consistent with those of Trentelman et al. [29], who reported strong inhibition of engorgement of *R. australis* larvae when fed in vitro on serum from animals immunized with a combination of Bm86 and Subolesin compared with larvae fed serum from animals immunized with these antigens individually.

In contrast, cattle immunized with 100 μg pBm86 + 50 μg pSubolesin as a co-immunization showed an overall efficacy of 50%, which is lower than that reported in previous controlled trials using these antigens under a single-antigen approach [6,9,10,19,24]. These results were consistent with those reported by Mendoza-Martínez et al. [11] under field conditions, indicating antigenic competition. In the context of antigenic competition, it has been observed that some epitope-associated antigens can show “immunodominance” over others, which are termed “immunosilent”. Several authors suggest that immunodominance may result from complex interactions among multiple factors. These include the efficiency of antigen processing, the stability and quantity of peptide-MHC II complexes expressed on antigen-presenting cells (APCs), the affinity and avidity of epitopes for MHC II and B-cell receptors (BCR), and the number of CD4+ T helper lymphocytes specific to each epitope [30,31]. This phenomenon leads to intra- or intermolecular competition among epitopes from the same or different antigens. It is dose-dependent and more pronounced when many antigens are present, resulting in intermolecular competition [14,16,32]. The findings of this study are consistent with previous research on sheep immunized with 4F8, 4D8 (Subolesin), and 4E6; each antigen was administered at 50 μg in three doses against *I. scapularis*. The cocktail vaccine showed lower efficacy (58%) than individual administration of Subolesin (71%) [33]. Similar results were reported by Olds et al. [34], who used a multivalent vaccine composed of six antigens combined with Subolesin at a dose of 50 μg each, administered in three doses against *R. appendiculatus* in cattle. The animals developed higher levels of IgG antibodies against Subolesin than against the other antigens. These results suggest that Subolesin is an immunodominant antigen, regardless of whether it is combined with Bm86. Although this study does not determine whether Subolesin possesses all the characteristics previously described as defining it as an immunodominant antigen, further research is required to identify the specific features that contribute to its immunodominance.

Vaccine efficacy has also been associated with the number of doses. In this study, the 100 μg pBm86 + 50 μg pSubolesin groups received two doses. Interestingly, although Group 1 (co-immunization) developed higher IgG antibody levels against pSubolesin (2.7 OD) than Group 2 (1.7 OD), the cocktail formulation induced higher anti-pBm86 antibody levels (1.1 OD vs. 0.7 OD) and resulted in markedly better vaccine efficacy (75% vs. 50%, respectively). This dissociation between antigen-specific IgG magnitude and overall vaccine efficacy suggests that protective performance may depend not only on antibody levels but also on the balance between immune responses directed against complementary antigens, as well as on antigen accessibility and functional relevance within tick tissues. In this context, the administration of a third booster could be explored to further optimize immune balance and protective efficacy. Notably, this observation contrasts with previous studies reporting that two doses of Bm86 antigen were enough to induce sustained IgG levels and protection against *R. microplus* infestation under field conditions [35,36,37].

The Western blot analysis indicated that the formulations used in this study are immunogenic. The anti-pSubolesin antibodies demonstrated a strong ability to bind to the salivary glands and ovaries of the *R. microplus* tick, although their detection in the gut was low. On the other hand, the anti-Bm86 antibodies showed strong recognition of the native protein in the gut; however, Group 1 revealed lower recognition in the ovaries. These findings are consistent with other experiments that reported that these antigens are immunogenic and can induce protective antibodies [10,11,15,22].

As discussed previously, temperature, humidity, and rainfall are key environmental factors influencing the establishment, survival, distribution, and growth of tick populations under field conditions [38]. This study recorded climatic variables monthly, showing patterns consistent with previous reports from this region [21,27]. A gradual increase in tick infestation was observed from June to August, peaking in September and remaining elevated through the end of the experiment. These findings are similar to those of some trials on natural tick infestation conducted in tropical regions [39,40]. It has been noted that seasonal or environmental changes could affect infestation levels in immunized cattle, influencing their immune response [7,41]. However, in the current study, tick infestations were consistently lower in immunized animals than in control cattle throughout the experimental trial. Moreover, no statistically significant correlations were detected between tick infestation levels and the climatic variables analyzed. Overall, these findings suggest that the observed reduction in tick infestation may be due to the cattle’s immune response rather than environmental fluctuations.

In summary, the results of this study suggest that antibodies induced by pBm86 and pSubolesin act through distinct yet complementary mechanisms in tick biology, thereby enhancing vaccine efficacy when combined. While anti-pBm86 antibodies have been shown to disrupt the intestinal epithelium, leading to impaired gut function, reduced tick weight, and decreased oviposition [6,15], anti-pSubolesin antibodies affect ticks’ salivary glands and ovaries, reducing feeding efficiency and reproductive capacity [8,9]. In this study, the cocktail formulation of 100 μg pBm86 + 50 μg pSubolesin showed the highest effect on tick biological parameters. Therefore, combining these antigens at appropriate concentrations may have a synergistic effect on the feeding and reproduction of *R. microplus* compared with a single-antigen approach. However, further studies are required to test this strategy in greater detail, particularly to optimize antigen concentrations, formulation design, and vaccination schedules, in order to validate our hypothesis. Such studies will be necessary to identify the optimal antigen combination and immunization scheme that enhances protective efficacy across diverse field conditions.

## 5. Conclusions

In conclusion, this study identified a promising formulation combining the pBm86 and pSubolesin antigens to control *R. microplus* in naturally infested cattle. The formulation consisting of 100 μg pBm86 + 50 μg pSubolesin induced antigen-specific humoral responses, associated with a significant reduction in tick infestation parameters. In addition, vaccinated cattle developed higher IgG antibody levels against pSubolesin than pBm86, indicating that Subolesin may be an immunodominant antigen in this formulation. Overall, our findings support the strategic combination of pBm86 and pSubolesin to improve vaccine efficacy against *R. microplus* compared with previously reported single-antigen approaches. Finally, future research should focus on elucidating the factors that influence antigen immunodominance in order to design optimized multi-antigen, polypeptide-based vaccines as a promising and sustainable approach for the control of cattle tick infestations.

## Figures and Tables

**Table 1:** Effect of immunization with recombinant tick antigens (pBm86 and pSubolesin) on the control of *R. microplus* infestations in cattle.

*Rhipicephalus microplus*						
		Percent Reduction (Immunized/Control) ^b^				
Experimental Group ^a^		DT	DW	DO	LH	E ^c^
Group 1	100 μg pBm86 + 50 μg pSubolesin (co-immunization)	22% (208 ± 12)	7% (138 ± 7)	16% (51 ± 2)	23% * (24 ± 1)	50%
Group 2	100 μg pBm86 + 50 μg pSubolesin (cocktail)	59% * (110 ± 14)	11% (132 ± 20)	20% * (48 ± 8)	25% * (23 ± 3)	75%
Group 3	Adjuvant/saline control					
		(267 ± 23)	(149 ± 3)	(61 ± 4)	(31 ± 2)	---

^a^ Cattle were randomly assigned to experimental groups (N = 4), immunized, and challenged with *R. microplus* (field conditions). ^b^ The percent reduction was calculated with respect to the control group. In parentheses, the average ± SD for adult female tick number (DT), tick weight (DW), oviposition (DO), and larvae hatching (LH) are shown and were compared by Kruskal–Wallis test between immunized and control groups (* *p* < 0.05). ^c^ Vaccine efficacy (E) was calculated as 100 [l-(CRT × CR0 × CRF)], where CRT, CRO and CRF are the reductions in the number of adult female ticks, oviposition, and larvae hatching as compared to the control group, respectively.

## Data Availability

The original contributions presented in this study are included in the article/Supplementary Materials. Further inquiries can be directed to the corresponding author.

## Supplementary Materials

The following supporting information can be downloaded at https://www.mdpi.com/article/10.3390/vetsci13030301/s1: Figure S1: Capillary artificial feeding test; Figure S2: Temporal dynamics of *Rhipicephalus microplus* during immunization trial and its correlation with environmental variables; Table S1: Environmental variables recorded during immunization trial.

## Abbreviations

The following abbreviations are used in this manuscript:

ITM	Integrated tick management
masl	Meters above sea level
CEIEGT	Center for Teaching, Research and Extension in Tropical Livestock
FMVZ	Faculty of Veterinary Medicine and Zootechnics
UNAM	National Autonomous University of Mexico
PBS	Phosphate-buffered saline
ELISA	Enzyme-linked immunosorbent assay
Tw	Tween 20
OD	Optical density
AP	Alkaline phosphatase
SDS-PAGE	Sodium dodecyl sulfate–polyacrylamide gel electrophoresis
PVDF	Polyvinylidene fluoride
BCIP/NBT	5-bromo-4-chloro-3-indolyl-phosphate/nitro blue tetrazolium
CENID-SAI	Centro Nacional de Investigación Disciplinaria en Salud Animal e Inocuidad
INIFAP	Instituto Nacional de Investigaciones Forestales, Agrícolas y Pecuarias
SD	Standard deviation
CI	Confidence interval
MHC II	Major histocompatibility complex class II
APC	Antigen-presenting cell
BCRs	B-cell receptors
