# Supplementary material for: Exploring the Combined Effect of Bm86 and Subolesin Polypeptide Vaccines in Cattle Naturally Infested with Rhipicephalus microplus
Source: Vet Sci. 2026 Mar 22;13(3):301. doi: 10.3390/vetsci13030301 (PMC13030643; doi:10.3390/vetsci13030301)
Supplement: Supplementary file 1 [file vetsci-13-00301-s001.zip › FIGURE S1.pdf]

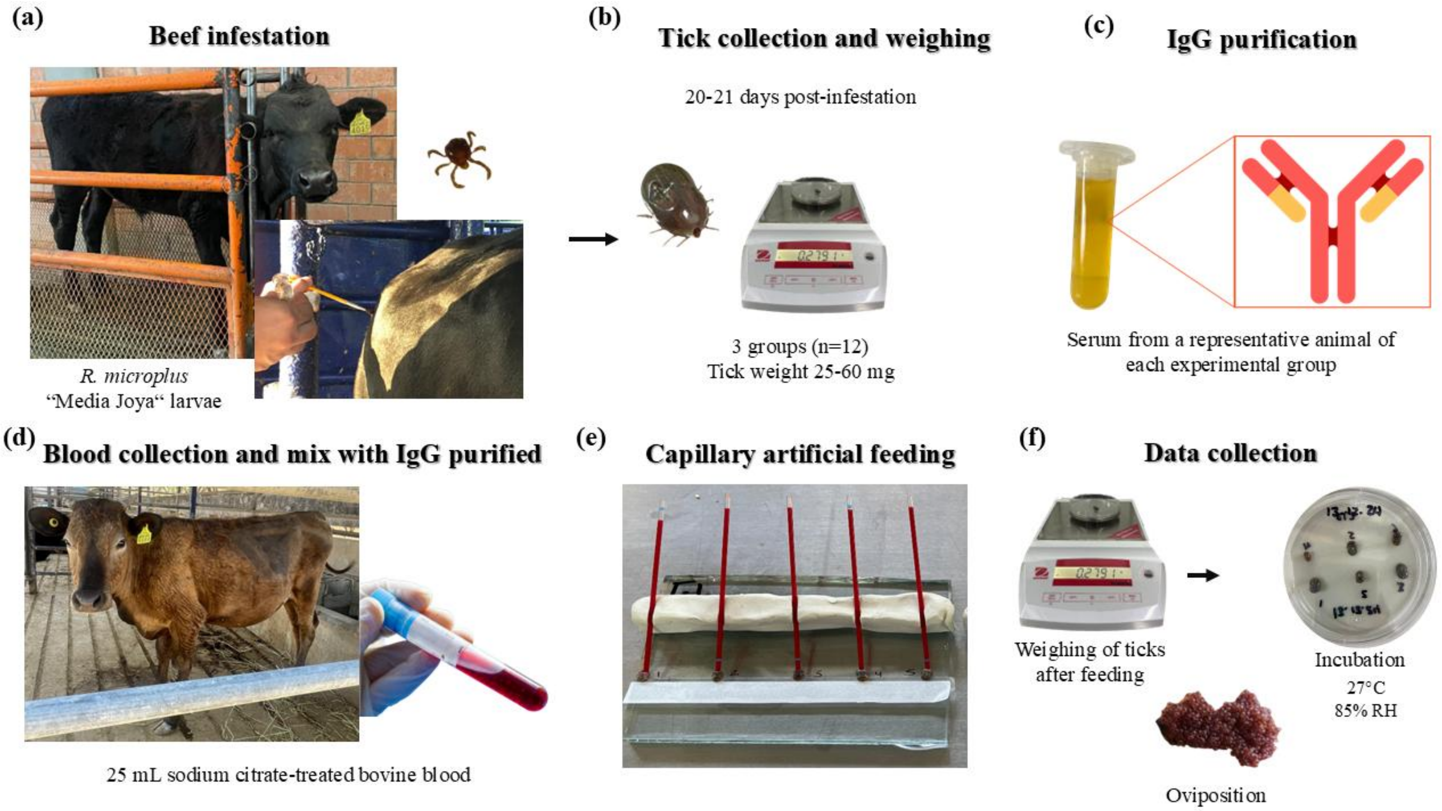

Figure S1. Capillary Artificial Feeding Test. **(a)** A *Bos taurus* calf was artificially infested with *R. microplus* larvae of the "Media Joya" strain. **(b)** 20-21 days after infestation, adult ticks were collected, weighed individually, and divided into three groups (N=12**)**. **(c)** 1 ml of IgG anti-Bm86 and anti-Subolesin was purified from the serum of a representative animal from each experimental group of the immunization assay. **(d)** 25 ml of blood was collected from a *Bos taurus* calf in tubes containing sodium citrate as an anticoagulant and mixed with the previously purified IgG. **(e)** The collected ticks were fed using capillaries for 48 hours. **(f)** The engorged ticks were weighed individually after the feeding period and incubated at 27°C and 85% RH to evaluate oviposition.
