# Supplementary material for: Exploring the Combined Effect of Bm86 and Subolesin Polypeptide Vaccines in Cattle Naturally Infested with Rhipicephalus microplus
Source: Vet Sci. 2026 Mar 22;13(3):301. doi: 10.3390/vetsci13030301 (PMC13030643; doi:10.3390/vetsci13030301)
Supplement: Supplementary file 1 [file vetsci-13-00301-s001.zip › FIGURE S2.pdf]

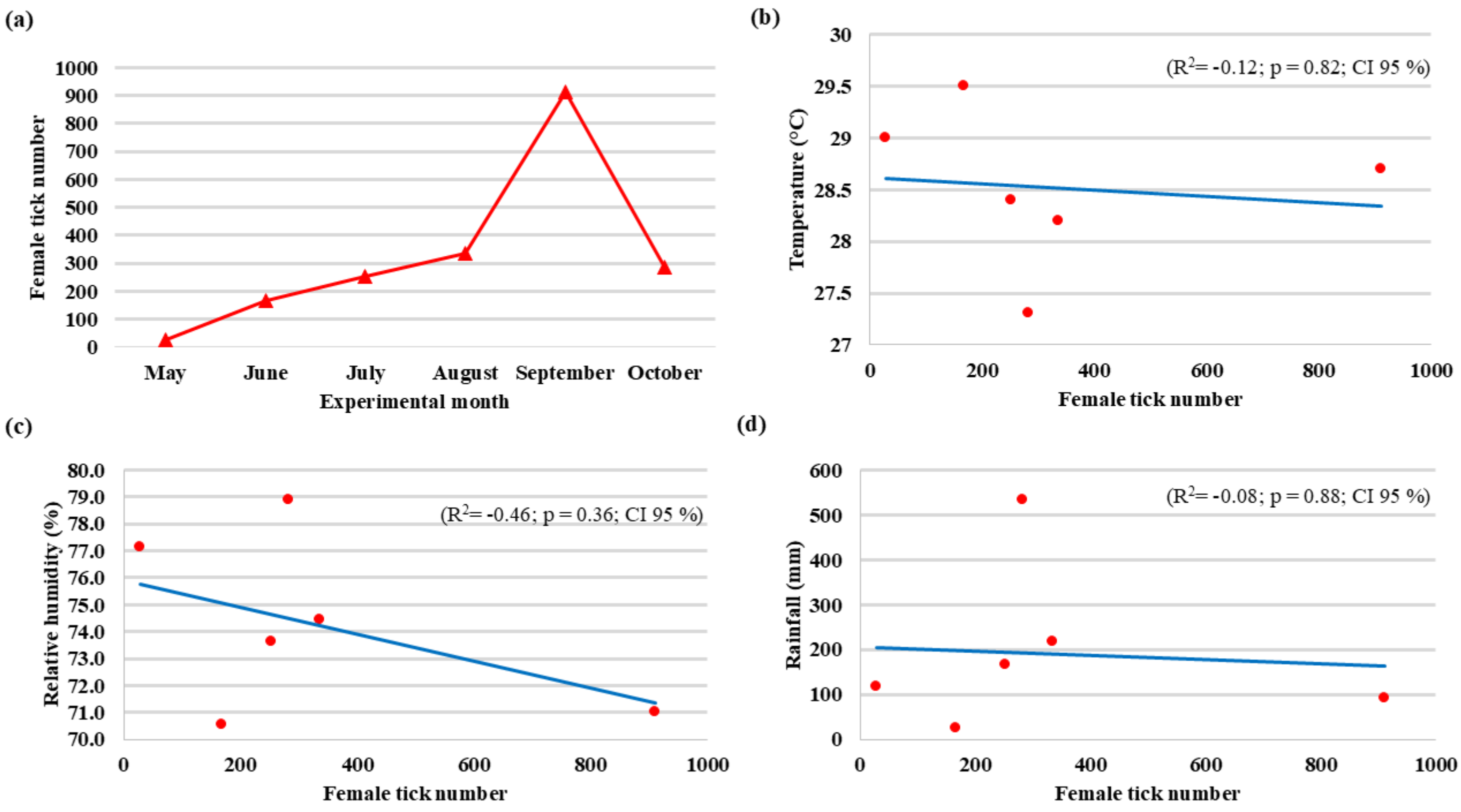

Supplementary Figure S2. Temporal dynamics of *Rhipicephalus microplus* during immunization trial and its correlation with environmental variables. (**a**): Total number of adult female ticks collected during the experimental trial. The Pearson's correlation analyses ($P < 0.05$) were conducted to correlate the adult female tick number collected during the experimental trial with the monthly environmental variables such as: (**b**) environmental temperature (◦C), (**c**) relative humidity [RH] (%), and (**d**) rainfall (mm). The linear correlation coefficients (R^2), p-value and confidence interval (CI) are shown.
