# Supplementary material for: Exploring the Combined Effect of Bm86 and Subolesin Polypeptide Vaccines in Cattle Naturally Infested with Rhipicephalus microplus
Source: Vet Sci. 2026 Mar 22;13(3):301. doi: 10.3390/vetsci13030301 (PMC13030643; doi:10.3390/vetsci13030301)
Supplement: Supplementary file 1 [file vetsci-13-00301-s001.zip › TABLE S1.pdf]

Table S1. Environmental variables recorded during immunization trial.

Month	Temperature (°C)	Relative Humidity (%)	Total rainfall (mm)*
	(Mean ± S.D.)		
May 2023	29.0 ± 1.3	77.1 ± 7.4	116.1
June 2023	29.5 ± 4.0	70.6 ± 10.0	23.9
July 2023	28.4 ± 2.6	73.7 ± 7.4	166.9
August 2023	28.2 ± 2.7	74.4 ± 6.6	216.4
September 2023	28.7 ± 2.3	71.0 ± 5.6	92
October 2023	27.3 ± 7.3	78.9± 7.1	533.2

The monthly average of environmental temperature (◦C), relative humidity [RH] (%), and total rainfall (mm) recorded in the experimental area (Rancho "El Clarín") during the trial. *Data obtained from the National Weather Service (2025) https://smn.conagua.gob.mx/tools/RESOURCES/Normales_Climatologicas/Mensuales/ver/mes30102.txt
